# Effects of Adding Zero Valent Iron on the Anaerobic Digestion of Cow Manure and Lignocellulose

**DOI:** 10.3389/fbioe.2020.590200

**Published:** 2020-10-28

**Authors:** Yu Men, Lei Zheng, Lingling Zhang, Zifu Li, Xuemei Wang, Xiaoqin Zhou, Shikun Cheng, Wenjun Bao

**Affiliations:** Beijing Key Laboratory of Resource-oriented Treatment of Industrial Pollutants, International Science and Technology Cooperation Base for Environmental and Energy Technology of Ministry of Science and Technology of People's Republic of China, School of Energy and Environmental Engineering, University of Science and Technology Beijing, Beijing, China

**Keywords:** anaerobic digestion, methane, zero valent iron, cow manure, lignocellulose

## Abstract

Previous studies showed that adding zero valent iron (ZVI) can increase the methane production and degradation rate of organic waste by improving the performance of anaerobic digester. However, our study firstly found that ZVI (37 μm, 10 g/L) inhibited the anaerobic digestion (AD) of cow manure and lignocellulose. ZVI significantly increased the methanogenic rate of cow manure in the first 6 days, but decreased the accumulative methane yield and volatile fatty acids yield by 10.3 and 12%, respectively. The effect of ZVI on AD of liquid biomass separated from cow manure was positive, but the effect on solid biomass was negative. These results indicated that ZVI enhanced the AD of easily biodegradable organics but inhibited the biodegradation of refractory organics (lignocellulose). By analyzing the varying effects of ZVI in diverse anaerobic systems, it was found that the effects were influenced by the characteristics of substrate and inoculum-substrate ratio. This study suggested that only proper ZVI addition can improve the AD process depending on the feeding materials.

## Introduction

Anaerobic digestion (AD), with the merits of reducing organic pollution, energy recovery, and low operation cost, has become a widespread pathway in converting organic waste to energy (Romero-Güiza et al., 2016). Agricultural solid waste, including livestock manure and crop straw, is regarded as a reused biomass resource due to its high content of biodegradable organic matters and nutrients (Lu et al., 2016). Among them, cow manure (one of the main livestock manure) contains not only easily degradable organics such as proteins, lipids, and soluble polysaccharides, but also refractory organics such as lignocellulose. As a major carbon source of agricultural solid waste in AD system, lignocellulose is difficult to be biodegraded because of its highly resistant and recalcitrant biomass structure (Sawatdeenarunat et al., 2015). To improve the biodegradability of substrate and the efficiency of AD, several approaches have been suggested and evaluated in many studies, such as mechanical or chemical pretreatment (Hendriks and Zeeman, 2009), co-digestion with several mixed substrates (Wang et al., 2018), and introduction of additives (Romero-Güiza et al., 2016).

Zero valent iron (ZVI)—a strong reductive material—is among the most prominent additive to enhance the AD of wastewater and waste activated sludge due to its non-toxicity, abundance, low cost, and easy manufacturing (Hwang et al., 2019). ZVI promotes the hydrogenotrophic methanogenesis by providing electrons or hydrogen evolution from the iron corrosion, which results in increased CH_4_ production from the consumption of CO_2_, as shown in Equations (1)–(3) (Hu et al., 2015; Xu et al., 2019).

(1) $${\text{CO}}_{2}+{\text{4Fe}}^{0}+{\text{8H}}^{+}\;\to \;{\text{CH}}_{4}+{\text{4Fe}}^{2}++{\text{2H}}_{2}\text{O}$$

(2) $${\text{Fe}}^{0}+{\text{2H}}_{2}\text{O }\to \;{\text{Fe}}^{2}++{\text{H}}_{2}+{\text{2OH}}^{-}$$

(3) $${\text{CO}}_{2}+{\text{4H}}_{2}\;\to \;{\text{CH}}_{4}+{\text{2H}}_{2}\text{O}$$

In addition, ZVI also accelerates the hydrolysis process of sludge by converting particulate matter to soluble substrates, and enhances the conversion of propionate to acetate (Meng et al., 2013). ZVI (20 g/L) increased the accumulative methane production by 43.5% of waste activated sludge, promoted the decomposition of protein and polysaccharide, and accelerated both methanogenesis and hydrolysis–acidification processes (Feng et al., 2014). However, a recent report suggested that ZVI has a positive effect on the AD of waste activated sludge, but has no, or little effect on the solubilization, hydrolysis, and acidification processes (Zhao et al., 2018). Moreover, the effect of microscale ZVI on the biochemical methane potential (BMP) of blackwater is negligible (Xu et al., 2019). Therefore, ZVI has a positive effect on AD of some organic wastes, especially waste activated sludge, and has diverse effects on AD in various systems.

Many studies have investigated the role of ZVI in the AD of various organic wastes, but the effect on cow manure is still unknown. In the anaerobic co-digestion process of cow manure and Phragmites straw, Fe^2+^ increased the accumulative biogas yield and methane content by 18.1 and 8.3%, respectively, and extended the gas production peak stage by improving the cellulase activities (Zhang et al., 2016). Compared with Fe^2+^, ZVI can decrease oxidative–reductive potential of the anaerobic digestion media, serve as electron donor for hydrogenotrophic methanogens, and provide a more favorable environment for anaerobic digestion. However, waste iron powder had no effect on digestion performances of cow manure (Andriamanohiarisoamanana et al., 2018). Therefore, it is inconclusive that whether ZVI can enhance the AD of cow manure and accelerate the hydrolysis, acidification and methanogenesis process.

This study investigated the effects of ZVI on the hydrolysis–acidification processes and methanogenesis of cow manure. Liquid and solid biomass separated from cow manure were used for BMP tests to study the mechanism of ZVI inhibition. Besides, this study investigated the effects of ZVI on AD of two model substrates (starch and cellulose) and cow manure under varying inoculum-substrate ratio (ISR). ZVI played diverse roles in different AD systems depending on the components of substrate, which was studied and summarized to provide a reference for the practical application of ZVI in AD system.

## Materials and Methods

### Materials

Cow manure and untreated inoculum were collected from the same dairy farm of Beijing, China. Waste activated sludge was collected from a municipal wastewater treatment plant of Beijing, China. The cow manure was stored in a sealed bag and placed in a refrigerator at −20°C before experiments. The inoculum was incubated with glucose for 2 weeks, and removed the supernatant before use. Waste activated sludge was stored in a serum bottle and placed in a refrigerator at 4°C before experiments. Microcrystalline cellulose (90 μm) and starch (AR) were purchased from Shandong Xiya Reagent Co., Ltd. and Sinopharm Chemical Reagent Co., Ltd., respectively. ZVI (purity > 98%, 37 μm in diameter) was purchased from Aladdin Reagent Co., Ltd., China.

Cow manure was washed with an equal weight of deionized water and extruded by a sieve (0.2 mm) for solid–liquid separation. The separated liquid was labeled as liquid cow manure, and the separated solid was labeled as residue. The main characteristics of cow manure, liquid cow manure and residue are listed in Table 1. The contents of total solid (TS) of untreated inoculum, inoculum, and waste activated sludge were 4.96, 4.44, and 4.84%, respectively. The contents of volatile solid (VS) of untreated inoculum, inoculum, and waste activated sludge were 55.64, 53.14, and 51.18%, respectively. The content of total soluble iron of inoculum was 2.29 mg/L.

**Table 1:** Characteristics of cow manure, liquid cow manure, and residue.

**Parameter**	**Cow Manure**	**Residue**	**Liquid cow manure**
TS (g/100 g)	20.95	30.04	3.45
VS (% TS)	78.65	85.73	57.50
pH	ND	ND	7.63
Carbon (% TS)	35.05	35.34	39.22
Nitrogen (% TS)	2.01	0.96	5.35
C/N	17.44	36.81	7.33
Starch (% TS)	9.51 ± 0.28	8.69 ± 0.19	ND
Cellulose (% TS)	24.88 ± 0.37	31.23 ± 0.28	ND
Hemicellulose (% TS)	15.80 ± 0.27	22.27 ± 0.63	ND
Lignin (% TS)	10.93 ± 0.04	15.35 ± 0.14	ND
Total soluble iron (mg/L)	10.69	3.97	1.49
Total mineral iron (mg/kg TS)	1532.4	1986.5	ND

*Values are expressed as mean ± standard deviation (n = 3)*.

*TS, total solids; VS, volatile solids; ND, not determined*.

### Experimental Design

The effects of ZVI on AD of several substrates were investigated using BMP tests. BMP tests were conducted by an Automatic Methane Potential Test System II (Bioprocess Control AB, Sweden), as described below. The mixture (VS_inoculum_:VS_substrate_ = 2:1) of substrate and inoculum in a 500 mL serum bottle (working volume = 400 mL) was placed in a water bath at 36 ± 1°C. All serum bottles were sealed with butyl rubber stoppers after flushing with nitrogen, and stirred for 1 min every 5 min at 120 rpm by an automatic stirring rod. A serum bottle (working volume = 80 mL) containing 3M NaOH was connected to the reactor through a natural latex tube to fix CO_2_. Biogas volume (without CO_2_) was automatically recorded after entering the gas volume measuring device through a natural latex tube.

ZVI particles were added to reach the final concentration of 10 g/L in the 500 mL serum bottle, which was selected on the base of the effective dosage range in literature. Each batch experiment included a control (without ZVI, labeled as C) and a ZVI addition group (labeled as Z). Six sets of BMP tests under ISR of 2 were set up as follows: cow manure (C-CM1, Z-CM1), liquid cow manure (C-LCM, Z-LCM), residue (C-R, Z-R), microcrystalline cellulose (C-MC, Z-MC), starch soluble (C-S, Z-S) and cow manure inoculated with untreated inoculum (C-UI, Z-UI). To explored the effects of ZVI on the hydrolysis–acidification of cow manure, 50 mM sodium 2-bromoethanesulfonate (SBES) was added in C-CM2 and Z-CM2 to remove methanogens (Feng et al., 2014), and the mass ratio of substrate to inoculum was fixed at 1:1 (on the basis of VS). All trials were conducted in triplicate.

### Chemical Analysis

TS and VS were determined by differential weighing after drying at 105 °C overnight and by subsequent incineration at 550°C, respectively, according to standard methods. pH was directly determined with a pH meter (HQ30d, Hach, USA). Cellulose, hemicellulose, and lignin contents of cow manure and residue were determined by Van Soest. Starch content was determined by the sulfonic acid ketone method. Carbon and nitrogen contents in dried biomass materials were detected by an elemental analyzer (vario EL cube, Elementar, Germany). For determination of the total metallic iron, cow manure and residue were digested with HNO_3_, then the digested liquid was filtered by 0.45 μm membrane and quantified for total soluble iron. Liquid samples were centrifuged at 8,000 rpm for 20 min at 4°C and filtered through a 0.45 μm polyether sulfone membrane to quantify the soluble product. The concentration of total soluble iron was measured by inductively coupled plasma-optical emission spectroscopy (725 ES, Agilent, USA). Soluble polysaccharide and soluble proteins were measured by using phenol–sulfuric acid method and Bradford Protein Assay Kit, respectively, with a microplate spectrophotometer (SpectraMax Plus^384^, Molecular Devices, USA). Volatile fatty acids (VFAs) were analyzed by a gas chromatograph (GC-8600, Beijing) equipped with a flame ionization detector. The operating temperatures of the oven, injection port, and detector were 130, 220, and 250°C, respectively. The injection volume was 1 μL, and He was the carrier gas. The composition of biogas was analyzed using the same gas chromatograph equipped with a thermal conductivity detector. The temperature of the detector was 70°C.

### Statistical Analysis

SPSS (SPSS 24.0) was used for *t*-test analysis and *p* < 0.05 was considered to be statistically different.

### Data Analysis

The theoretical moles of methane for each mole of the substrate was calculated by the following stoichiometric equation (Buswell and Mueller, 1952):

(4) $$\begin{array}{l} C_{n}H_{a}O_{b}N_{c}+\left(n-\frac{a}{4}-\frac{b}{2}+\frac{3c}{4}\right)\;H_{2}O\\ \;\to \left(\frac{n}{2}+\frac{a}{8}-\frac{b}{4}-\frac{3c}{8}\right)\;CH_{4}\\ \;+\left(\frac{n}{2}-\frac{a}{8}+\frac{b}{4}+\frac{3c}{8}\right)\;CO_{2}+cNH_{3} \end{array}$$

The theoretical methane potential (*Y*_*Th*_, mL/g VS) of the substrate was calculated using the theoretical moles of methane by the ideal gas law, as shown in Equation (5):

(5) $$\begin{array}{l} \;Y_{Th}=\frac{22.4\times 1000\times \left(\frac{n}{2}+\frac{a}{8}-\frac{b}{4}-\frac{3c}{8}\right)}{12n+a+16b+14c} \end{array}$$

The level of anaerobic biodegradability (*BD*_*C*_*H*__4__, %) was calculated by the accumulative methane yield (*Y*_*Exp*_,mL/g VS) under the BMP test in comparison with its theoretical value as follows (Elbeshbishy et al., 2012):

(6) $$\begin{array}{l} BD_{CH_{4}}=\left(Y_{Th}/Y_{Exp}\right)\times 100 \end{array}$$

## Results and Discussion

### Effects of ZVI on the AD of Cow Manure

**Figure 1:**
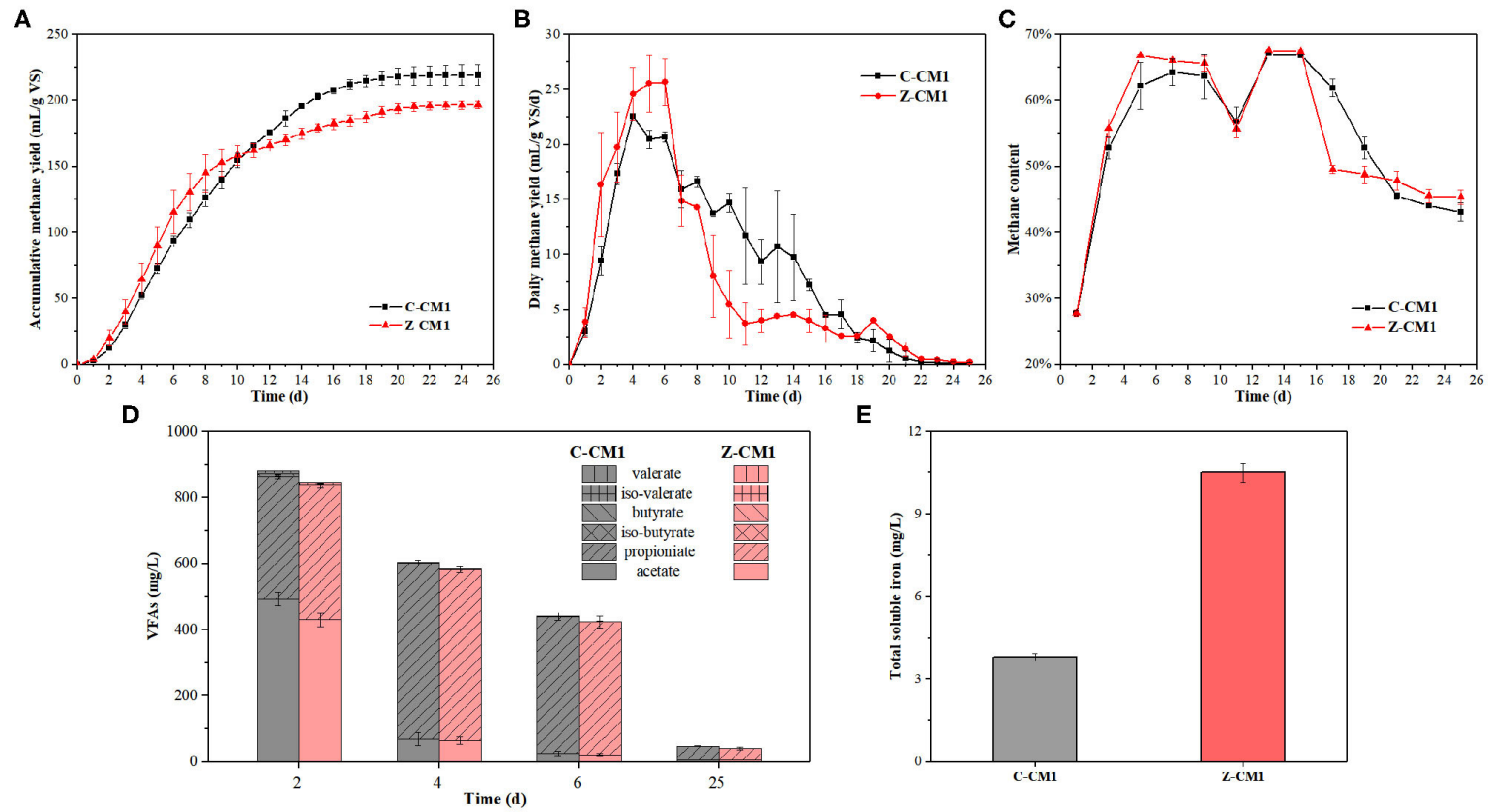
Changes in accumulative methane yield **(A)**, daily methane yield **(B)**, methane content **(C)**, and concentration and composition of VFAs **(D)** in AD of cow manure by BMP tests. **(E)** The content of total soluble iron in two reactors on day 25. C-CM1: reactors (500 mL) without ZVI; Z-CM1: reactors (500 mL) with 10 g/L ZVI.

During the 25 days digestion, biogas and supernatant were measured every day to analyze the effect of ZVI on AD of cow manure. Figure 1A shows the changes in accumulative methane. Both digesters started to produce methane quickly after a short period of adaptation, and the accumulative methane yield increased quicker in Z-CM1 than C-CM1 during the first 10 days. Compared with the control group, ZVI increased the methane yield in the initial stage, but gradually decreased it in the subsequent stages. After digestion, the groups treated with 10 g/L ZVI had significantly lower accumulative methane yield than the control, which were 196.6 ± 2.9 mL/g VS and 219.2 ± 7.8 mL/g VS, respectively. This result was inconsistent with the previous studies that ZVI played an active role and increased the methane production in some AD system (Hao et al., 2017; Kong et al., 2018). However, cow manure was not used as a substrate in previous studies, which might be the reason for the difference. There existed two methanogenesis periods (days 1–7 and days 7–25) of the daily methane yield (Figure 1B). During the first period, the higher daily methane yield was observed in Z-CM1. Then, the daily methane yield of Z-CM1 rapidly decreased from day 7 and remained at a much lower value than that of C-CM1 till the end. ZVI enhanced the methanogenic process of cow manure in first few days but reduced methane yield in following days and played a negative role. Easily degradable organics produced CH_4_ in the first period, and poorly biodegradable organics contributed to CH_4_ production in the second period (Zhang et al., 2019). Cow manure is a mixture of organic components with varying biodegradability, and contains plenty of lignocellulose (516.1 g/kg TS), which is difficult to be biodegraded. Thus, ZVI had opposite effects during the two periods probably because ZVI facilitated the AD process of easily degradable organics in cow manure but inhibited the AD process of refractory organics.

The VFAs concentrations of digestate from both groups are shown in Figure 1D. Acetic acid and VFAs concentration in C-CM1 on day 2 were 492.6 and 880.8 mg/L, respectively, which were higher than those of Z-CM1 with 429.1 and 845.9 mg/L, respectively. The lower VFAs concentration in Z-CM1 indicated that ZVI facilitated the conversion of VFAs by improving the metabolic activity of anaerobic microbes (Yuan et al., 2020). Subsequently, VFAs concentration dropped as methanogen consumed acetic acid, and ZVI had no significant effect on the formation of acetic acid and VFAs at the same time (*p* > 0.05). During the AD of cow manure, VFAs concentration was always at a lower level compared with that in other studies, and remained far below the inhibitory levels, which indicated that methanogens were sufficient in both groups to convert acetic to CH_4_ in time, thus hydrolysis process was the rate-limiting step in AD of cow manure. ZVI had no effect on VFAs production from blackwater (Xu et al., 2019), protein, and carbohydrates (Zhao et al., 2018) but effectively increased the total VFAs yield from swine manure (Yang et al., 2018) and waste activated sludge (Feng et al., 2014). Therefore, the effect of ZVI on VFAs concentration in AD system associated with the composition of substrate and the organic loading rate of reactor. Most of the iron introduced into the anaerobic system is not readily accessible to microorganisms. Therefore, adding iron to iron-deficient digestion system can increase soluble iron content, which can increase the growth and metabolism of microorganisms (Cai et al., 2018). It could be observed from Figure 1E that the total soluble iron in Z-CM1 at significantly higher compared to that in C-CM1 at the end of the digestion, due to the iron corrosion. As the digestion progressed, available iron was gradually released from organic matters decomposition and adsorbed by intracellular enzymes in microorganisms or by suspended solids and microbial surfaces (Cai et al., 2019). The final total soluble iron of C-CM1 was 3.80 ± 0.13 mg/L, higher than the original total soluble iron at the first day. These results showed that the soluble iron was enough in this system for microorganisms and enzymes, as ingested iron from animal feed was excreted in cow manure (Yang et al., 2018), which again confirmed that the effect of ZVI in AD system was affected by the characteristics of the substrate.

Figure 1C shows the methane content of biogas in two groups. The methane content rapidly increased at the beginning, and reached the first peak on day 2, followed by a transient drop on day 11, and rebounded to the second peak on day 13. The reactors with ZVI addition had higher methane content of biogas than the control in the first 9 days. Methane was either produced via the cleavage of acetate (aceticlastic pathway) or by the reduction of CO_2_ with hydrogen (hydrogenotrophic pathway) and from methylated C1 compounds to a minor extent. ZVI sustained a high syntrophic hydrogenotrophic methanogenesis activity (Yang et al., 2018), which contributed to the high utilization efficiency of CO_2_ to produce CH_4_, thereby resulting in a high methane content. Zhao et al. (2018) also confirmed that ZVI remarkably promoted hydrogenotrophic and syntrophic methanogenesis. The dominant metabolic pathway for CH_4_ production at the startup phase was hydrogenotrophic methanogenesis, and then turned into aceticlastic methanogenesis in the latter steady phase (Huang et al., 2017). Therefore, ZVI increased the methane content at the early stage by enhancing the hydrogenotrophic methanogenesis.

### Effect of ZVI on the Acidification Phase of Cow Manure

**Figure 2:**
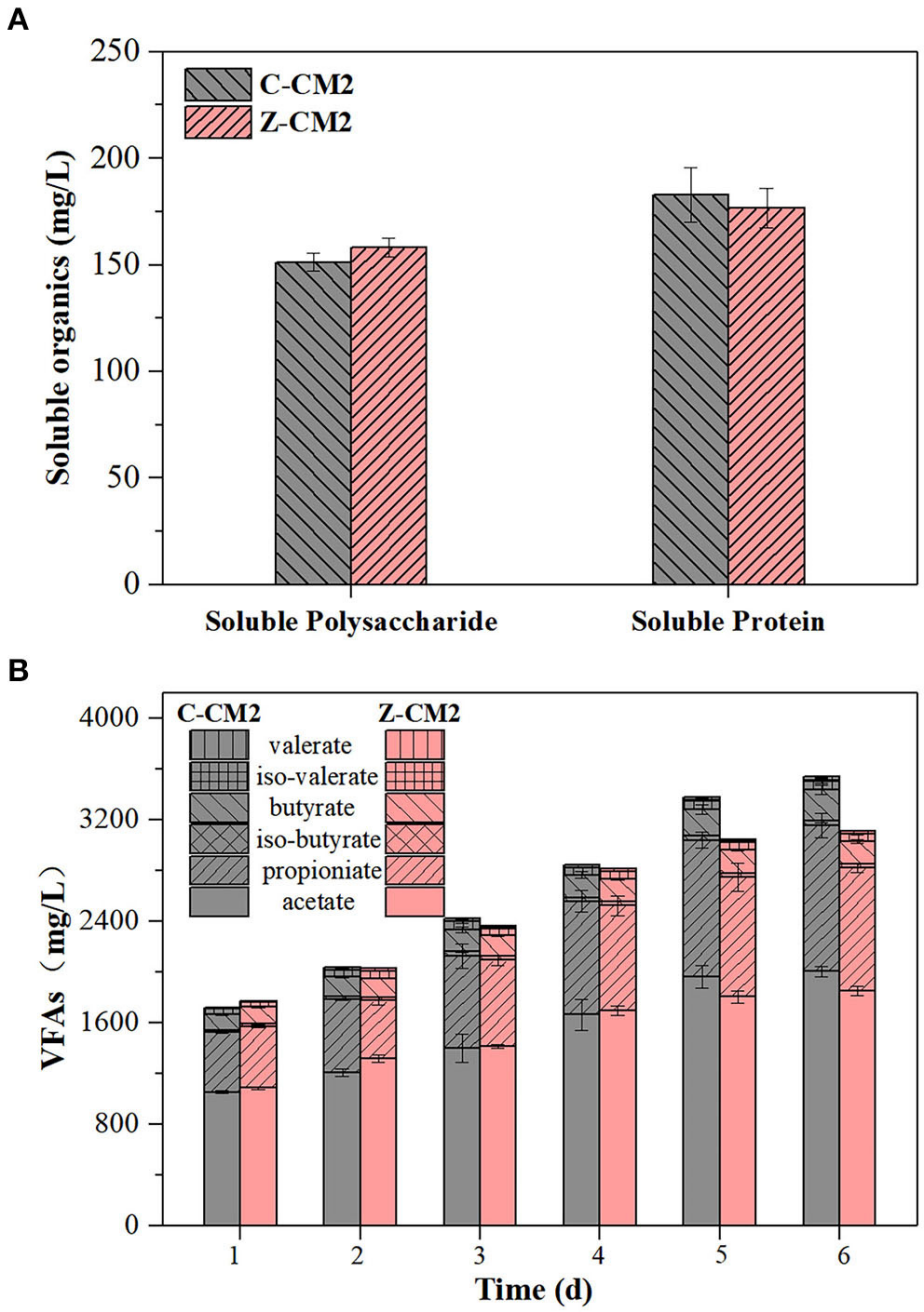
Effect of ZVI on the acidification phase of cow manure with SBES: **(A)** the soluble protein and polysaccharide concentrations on day 2; **(B)** the concentration and composition of VFAs. C-CM2: reactors (500 mL) without ZVI; Z-CM2: reactors (500 mL) with 10 g/L ZVI.

During the acidification process, complicated organics were first hydrolyzed into simpler and soluble organic compounds and then converted to VFAs by the biotransformation process referring to acidogenesis (De La Rubia et al., 2009). The decomposition rate of protein and polysaccharide typically limited the efficiency of AD. Thus, the concentrations of soluble protein and polysaccharide were measured to assess the solubilization of cow manure. Figure 2A shows that the concentrations of soluble protein and polysaccharide in Z-CM2 represented no significant difference compared with the control. The low concentrations of soluble protein and polysaccharide in both groups indicated that most of easily degradable organics were hydrolyzed into VFAs after 2 days. Under the addition of SBES, ZVI had no effect on the hydrolysis of protein and polysaccharide from easily degradable organics. Consistent with this study, ZVI had no or minimal effect on the hydrolysis process of bovine serum albumin and dextran, and drove hydrolysis forward in thermodynamics by enhancing methanogenesis (Zhao et al., 2018).

The most crucial product in the acidification phase is VFAs, because it is converted to acetate, H_2_, and CO_2_, which can further serve as a direct nutrient source for methanogenesis (Lu et al., 2018). SBES was added to reactors in order to prevent the VFAs from being consumed by methanogens. Figure 2B shows the concentrations of five types of VFAs, which contains acetate, propionate, iso-butyrate, butyrate, iso-valerate, and valerate. Total amounts of VFAs in two groups were basically non-distinctive in the first 4 days. Easily degradable substances (e.g., starch, proteins, and lipid) were degraded and converted into VFAs at this period. This result reconfirmed that ZVI had no or minimal effect on the hydrolysis process of easily degradable organics. The paired samples *t*-test indicated that ZVI significantly influenced the accumulation of VFAs on days 5 and 6 (*p* < 0.05). At the end of the experimental time, the VFAs concentration in Z-CM2 was 3539.8 mg/L, which was 12.0% lower than that of C-CM2. The higher accumulative VFAs production in Z-CM2 from day 5 indicated that ZVI suppressed the hydrolysis and acidification of refractory organic substances, such as lignocellulose. Therefore, ZVI decreased VFAs production by inhibiting the hydrolysis–acidification processes of cow manure, thereby decreasing the accumulative methane production.

### Effects of ZVI on the AD of Substrates With Different Biodegradability

**Figure 3:**
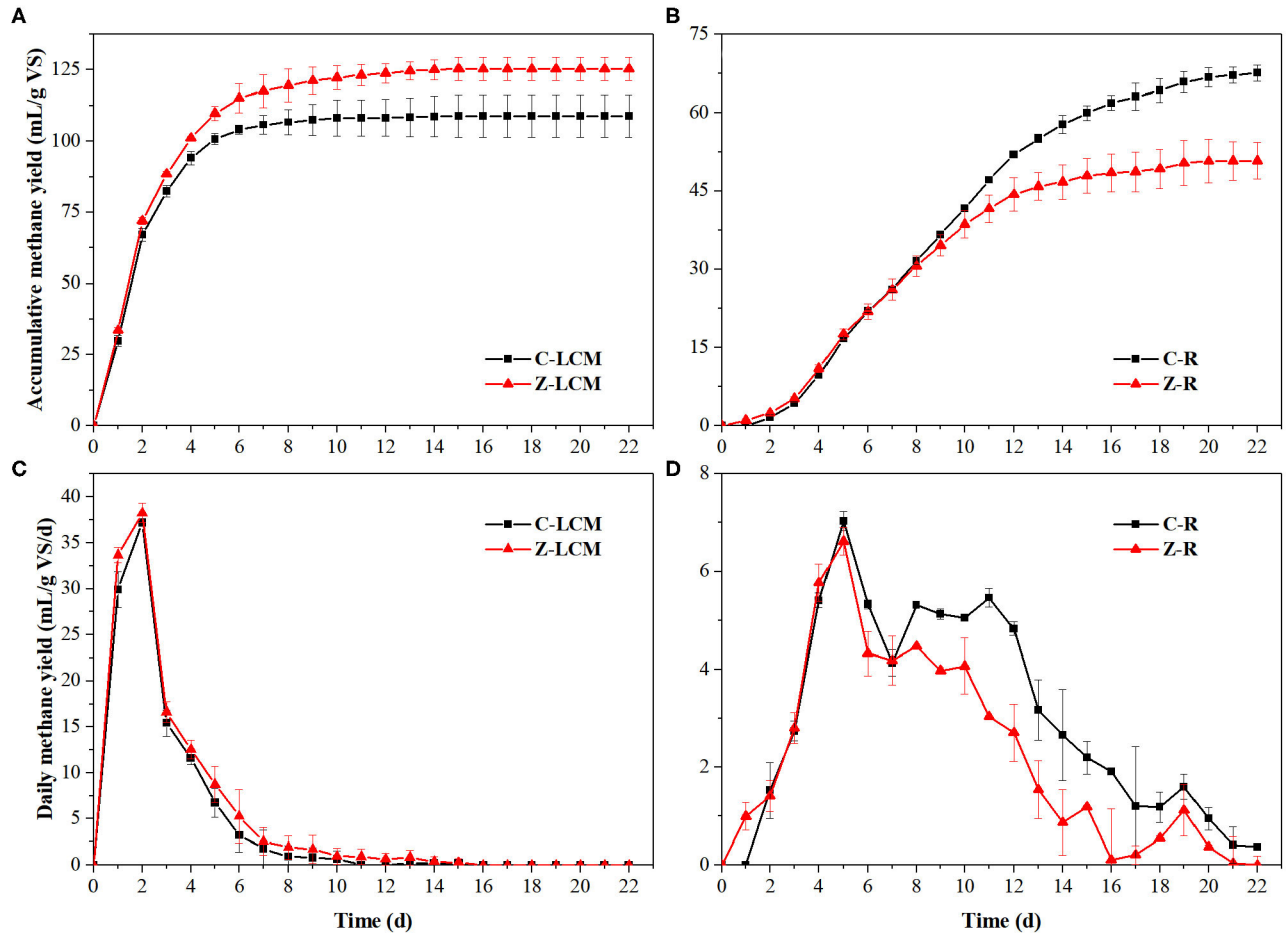
Changes in accumulative methane yield **(A,B)** and daily methane yield **(C,D)** in AD by BMP tests. C-R, AD of residue (solid part from solid–liquid separation of cow manure) without ZVI; Z-R, AD of residue with 10 g/L ZVI; C-LCM, AD of liquid cow manure (liquid part from solid–liquid separation of cow manure) without ZVI; Z-LCM, AD of liquid cow manure with 10 g/L ZVI.

According to the analysis of anaerobic performance in four reactors, ZVI had different effects on AD of various organics due to their varying biodegradability. Therefore, cow manure was separated to liquid part (liquid cow manure) containing easily degradable organics (e.g., protein, lipid, and soluble organics) and solid part (residue) containing refractory organics for BMP tests. Lignocellulose was the main refractory organic component of cow manure in this study—up to 51.61%, and difficult to be degraded due to its complex structure (Wang et al., 2018). Figure 3 shows the changes in accumulative methane yields and daily methane yields of liquid cow manure and residue during 22 days. ZVI showed a sustained promotion or inhibition effect on the AD of liquid cow manure and residue, respectively. The accumulative methane yield of liquid cow manure (Z-LCM) reached 125.4 ± 7.4 mL/g VS, which was 15.2% higher than that of C-LCM. And the accumulative methane yield of residue (Z-R) was 50.8 ± 3.5 mL/g VS, which was 20.4% lower than that of C-R. The higher methanation rate of liquid cow manure indicated that it was more accessible for the microorganisms to degradation than the residue. Because unbalanced C/N ratio of two substrates and inefficient anaerobic systems, the accumulative methane yields of both groups were lower than that of cow manure. Besides, ZVI had no significant influence on the AD of residue in the first few days but gradually decreased the accumulative methane yield from day 7, which suggests that ZVI mainly inhibited the degradation of residue at the later stage.

Microbes needed several days to decompose the lignocellulose into monomers, whereas the duration of hydrolysis of soluble carbohydrates was only a few hours. From Figure 3C, the daily methane yields of C-LCM and Z-LCM rapidly increased and reached maximum methane yield rate on day 2, respectively, then rapidly decreased to below 1 mL/g VS/day. Z-R began to produce biogas on day 1, whereas C-R started on day 2, which suggested that microorganisms in Z-R adapted to the environment better. The daily methane yields of residue were much lower than that of liquid cow manure, and reached the first peak of 7.0 ± 0.2 and 6.6 ± 0.9 mL/g VS/day on day 5, then reached the second peak on days 9–12. In the two methanogenesis stages, the difference between Z-R and C-R was negligible in the first stage but C-R performed better in the second stage, which indicated that ZVI mainly inhibited the AD of lignocellulose in residue.

ZVI decreased the methane yield of residue in this study, but Fe^3+^ showed no effect on methane yield of rice straw (Mancini et al., 2019) and Fe^2+^ increased the methane yield of another rice straw (Cai et al., 2017). As another major agriculture solid waste, rice straw also contained lots of lignocellulose, up to 63.13% (Cai et al., 2017). The reason for the difference might be related to different particle sizes: rice straw was cut down to a particle size smaller than 4 mm (Mancini et al., 2019); another rice straw was pulverized with a high-speed grinder and passed through a 1 mm mesh filter (Cai et al., 2017). Because milling (cutting the lignocellulosic biomass into smaller pieces) decreased particle size and crystallinity, which increased the available specific surface of substrate and decreased the degree of polymerization, contributing to an increase in hydrolysis yield of lignocellulose by 5–25% in most cases (Hendriks and Zeeman, 2009). Methane production was increased as the hydrolysis–acidification process was enhanced. Therefore, the methane production decrement could be offset by decreasing the particle size or promoting the hydrolysis. These results suggested that complex lignocellulosic biomass required an enhancement of the hydrolysis process by pretreatment rather than ZVI alone. For example, alkali treatment can destroy the structure of lignocellulose, thereby making it easier to be decomposed, which is beneficial to the digestion with Fe (Khatri et al., 2015).

### BMP Tests of Model Substrates

**Figure 4:**
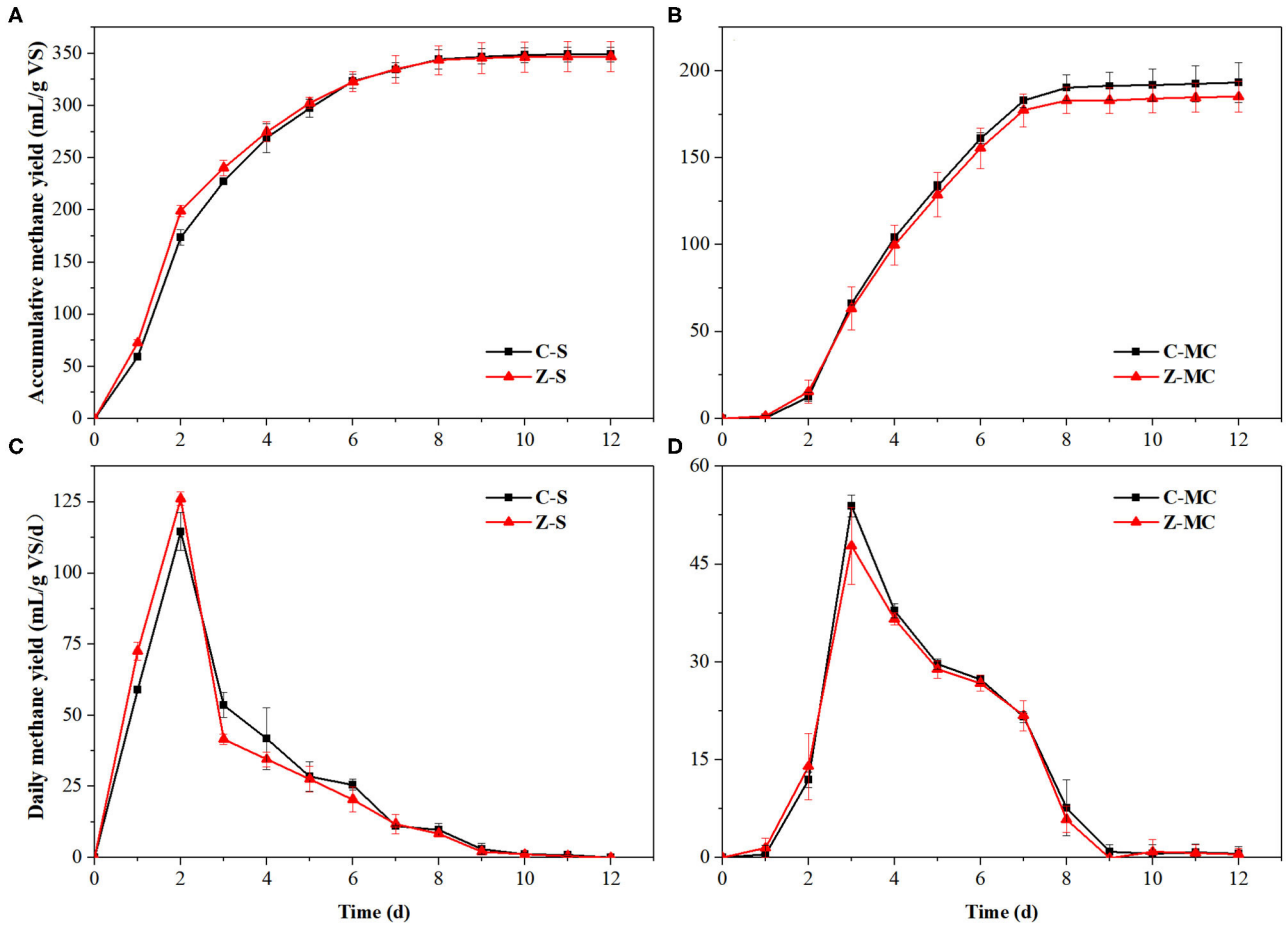
Changes in accumulative methane yield **(A,B)** and daily methane yield **(C,D)** in AD by BMP tests. C-MC, AD of microcrystalline cellulose without ZVI; Z-MC, AD of microcrystalline cellulose with 10 g/L ZVI; C-S, AD of starch without ZVI. Z-S, AD of starch with 10 g/L ZVI.

The main carbon sources in the residue were starch and lignocellulose, with the contents of 8.69 and 68.85%, respectively. Because ZVI had no effect on AD of residue in the beginning, and had negative effect on AD of lignocellulose at a later stage, starch—the uppermost renewable carbon source in residue besides lignocellulose—was used as a substrate for BMP test. Starch was synthesized in semi-crystalline granular structures polymerized by glucose, which was easily to be degraded (Wang et al., 2016). Figure 4 shows that the accumulative methane yield of Z-S was slightly higher than that of control in the first 2 days, and the daily methane yield of Z-S was significantly higher than that of C-S on day 2. This happened because ZVI promoted hydrogenotrophic and syntrophic methanogenesis, thereby increasing the rate of methanogenesis (Zhao et al., 2018). The daily methane yield of two groups began to drop from day 3 with the consumption of substrate, and then Z-S produced lower daily methane yield than control due to the faster consumption of substrate at the beginning. As shown in Figure 4A, there is no significant difference between the accumulative methane yields of Z-S and C-S, that is, ZVI had no effect on the BMP of starch, resulting in the alike accumulative methane yield of Z-R and C-R in the first few days (Figure 2A).

Lignocellulose primarily consists of cellulose, hemicellulose, and lignin. Among them, cellulose is composed of polymerized glucose molecules and has the highest organic carbon level in the residue (31.23%). Compared with lignin and xylose, cellulose could be degraded to produce a considerably higher accumulative methane production per gram, and contributed to most methane production in lignocellulose (Wyman et al., 2019). Both starch and cellulose are polysaccharides of glucose, with the same chemical formula—(C_6_H_10_O_5_)_n_, whose theoretical methane potential was 414.81 mL/g VS. According to **Equation (6)**, the level of anaerobic biodegradability of starch was 84.19%, which is much higher the cellulose of 46.59%. Daily methane yield in Z-MC was 6.0 mL/g VS/day on the first day, whereas it was 0.9 mL/g VS/day in C-MC, demonstrating that ZVI could shorten the start-up process of AD. The comparison of accumulative methane yield in Z-MC and C-MC showed that ZVI had no effect on the anaerobic degradation of cellulose. Microcrystalline cellulose was almost completely degraded on day 9, whereas the daily methane production in Z-R was gradually lower than that of C-R from day 8 (Figure 3D). These results suggested that ZVI mostly suppressed the disintegration and degradation of the recalcitrant lignocellulosic structure rather than cellulose. The lignocellulosic structure was formed by cross-linking among cellulose, hemicellulose, and lignin, which was a barrier for liquid penetration and enzyme access, thereby impeding the digestion of lignocellulosic biomass (Yang et al., 2015).

The disintegration and degradation of the lignocellulosic biomass was limited by several factors, including crystallinity of cellulose, available surface area, degree of polymerization, moisture content and lignin content (Hendriks and Zeeman, 2009). In anaerobic system, ZVI reacted with water to form hydrogen, which could simply be expressed by **Equation (7)**. As this reaction proceeded, the precipitation of Fe(OH)_2_ became favorable, and then Fe(OH)_2_ might be transformed to magnetite according to **Equation (8)** (Wei et al., 2018):

(7) $$\text{Fe}+{\text{2H}}_{2}\text{O }\to \text{ Fe}{\left(\text{OH}\right)}_{2}+{\text{H}}_{2}$$

(8) $$3\text{Fe}{\left(\text{OH}\right)}_{2}\to {\text{Fe}}_{3}{\text{O}}_{4}+{\text{H}}_{2}+{\text{2H}}_{2}\text{O}$$

Majority of nanoscale ZVI was precipitated in the form of FeOOH or iron oxide via the reaction with H_2_O (He et al., 2017). Any excess iron could precipitate as iron carbonate as long as the pH is 6.4 or above, and the pH values in all BMP tests was above 7.0 in this study. As ZVI was gradually corroded, Fe(II) and Fe(III) solids formed by ZVI would be adsorbed on the surface of lignocellulose, which reduced the available surface area of lignocellulose and the accessibility of cellulose, thereby inhibiting of the hydrolysis of lignocellulosic biomass.

### Effects of ZVI on the AD of Cow Manure Under Different Condition of Inoculum

**Figure 5:**
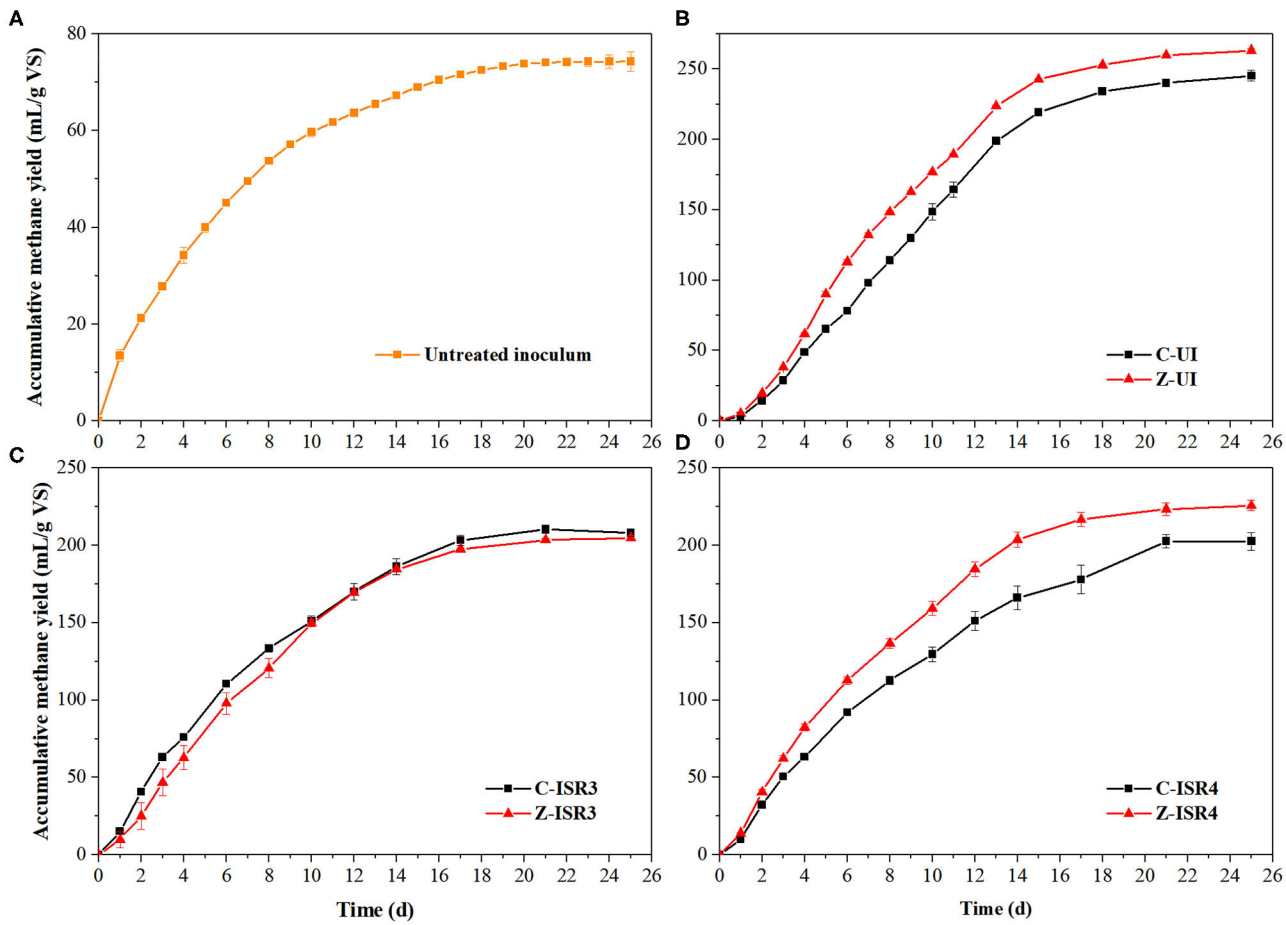
**(A)** Accumulative methane yield of untreated inoculum by BMP tests. And the effects of 10 g/L ZVI on accumulative methane yield of cow manure in various systems: **(B)** Inoculation with untreated inoculum; **(C)** ISR = 3; **(D)** ISR = 4.

Figure 5A shows the accumulative methane yield of untreated inoculum by BMP tests, which was collected from anaerobic fermentation tank in the dairy farm. The fresh inoculum was incubated at 36 ± 1°C for 2 weeks before use to consume excess substrate. The characteristic of inoculum is a crucial factor affecting the biochemical reactions during AD. As shown in Figure 5B, ZVI increased the accumulative methane yield of cow manure inoculated with untreated inoculum, which is contrary from the result described above. Because the anaerobic fermentation tank was feed with liquid cow manure, untreated inoculum contains more easily degradable organics. The increase of accumulative methane yield from easily degradable organics was higher than the decrease of accumulative methane yield from lignocellulose aroused by ZVI.

ISR is an important factor for the start of a balanced microbial population in anaerobic system, and an appropriate ISR is beneficial to balance the bacteria and archaea associated with the acidification and methanogenic process (Li et al., 2018; Zhang et al., 2020). A lower ISR means fewer methanogens and higher risk of VFAs inhibition, resulting in decreased biogas and methane yields (Latifi et al., 2019). A higher ISR can increases microbial populations and buffering capacity, but the excessive inoculum takes space and reduces the reactor utilization efficiency (Li et al., 2018). The effects of ZVI on the AD of cow manure under ISR of 3 and 4 were shown in Figures 5C,D, respectively. There was no significant difference among the accumulative methane yield of cow manure without ZVI under varying ISR of 2, 3, and 4, which indicated that there were enough microbes in three anaerobic systems to efficiently consume the substrates. At the same time, ZVI increased the accumulative methane yield of Z-ISR4 by 11.4%, and showed no effect on Z-ISR3, which meant that increasing ISR could alleviate even change the ZVI inhibition. With the increasing ISR, more microbes and easily degradable organics were introduced to anaerobic system, resulting in higher hydrolytic degree and biodegradability of total organics, thereby increasing the increment of methane production within methanogenesis process caused by ZVI.

### Comparison of the Effects of ZVI on the AD of Different Substrates

**Figure 6:**
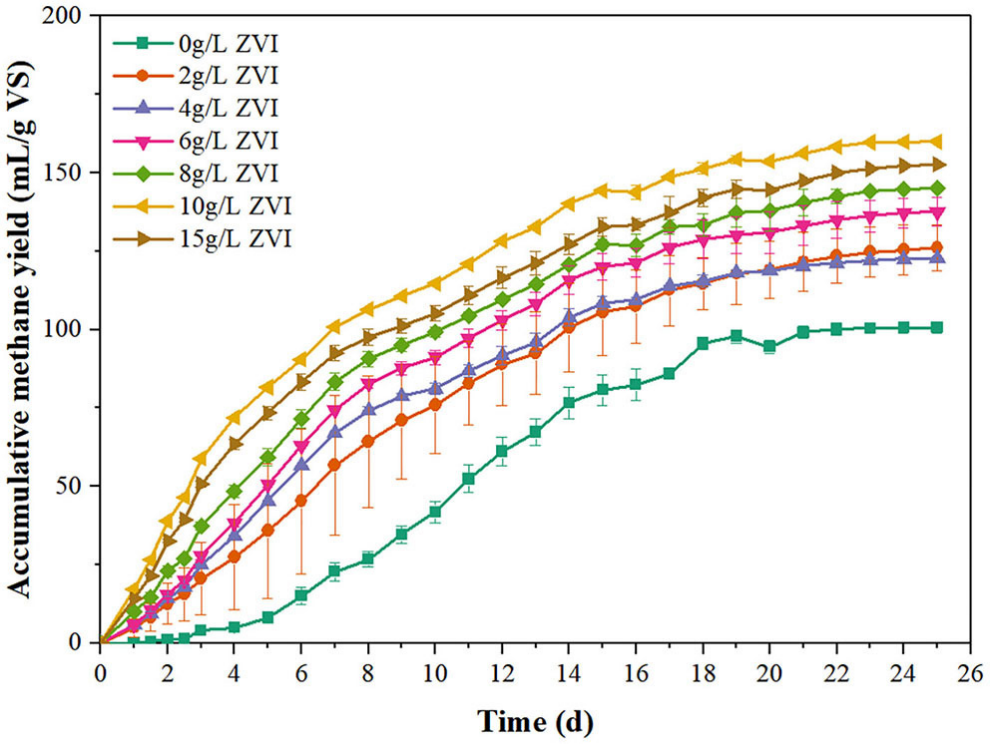
Accumulative methane yield from waste activated sludge during AD with and without ZVI by BMP tests.

Table 2 summarizes diverse effects of Fe on the AD system performance of various substrates. The influences of Fe^2+^ or Fe^3+^ was adopted because few studies investigated the effects of ZVI on AD of cow manure or lignocellulosic biomass. When the substrate was 30 g VS_substrate_/L organic fraction of municipal solid waste, reactors experienced a period of long-term excessive acidification from day 2.5, whereas ZVI could alleviate excessive acidification (Kong et al., 2018). The methane productivity of waste activated sludge at ZVI of 20 g/L increased by 43.5%, in which protein and polysaccharide accounted for 63.0 and 12.3% of organic matter, respectively (Feng et al., 2014). As shown in Figure 6, more (*p* < 0.05) methane was produced from waste activated sludge than the control during the whole period by adding ZVI at the studied six levels (i.e., 2, 4, 6, 8, 10, and 15 g/L), which was consistent with the results of waste activated sludge treatment in other studies (Feng et al., 2014; Zhao et al., 2018). The maximal accumulative methane yield of 160.0 ± 0.6 mL/g VS was achieved at ZVI dosage of 10 g/L, while only 100.7 ± 1.8 mL/g VS was produced from waste activated sludge without ZVI addition, representing a relative increase of 58.9%. If the substrate was much easily degraded, reactors could be overloaded with high organic loading rate, especially in batch systems, causing an imbalance between the acidogenesis/acetogenesis and methanogenesis steps, resulting in a large accumulation of VFAs. Substantial accumulation of VFAs decreased alkalinity and pH values, then inhibited methanogenesis (Braz et al., 2019). ZVI could accelerate the consumption of VFAs by enhancing methanogenesis, thereby driving hydrolysis and acidification forward in thermodynamics and increasing methane production (Zhao et al., 2018). Based on the previous reported studies, the mechanisms of iron enhancement on methanogenesis are summarized as three possible pathways: i) hydrogen evolution from iron corrosion could enhance hydrogenotrophic methanogenesis; ii) as an essential trace element for anaerobes, iron contributed to the growth of microbes responsible for CH_4_ production; iii) iron could stimulate the activities of enzymes involved in methanogenesis (Wei et al., 2018; Zhao et al., 2018). Therefore, with easily degraded substrate or high organic loading rates, ZVI could accelerate the conversion of excess VFAs to CH_4_, resulting in a stable and favorable condition for microorganism and methane production increase.

**Table 2:** Summary of the effects of Fe addition in different systems.

**Substrate**	**Type of iron**	**Dosage of Fe (g/L)**	**Methane yield (mL/g VS)**	**References**
Cow manure	ZVI	0 10	219.22 ± 7.83 196.64 ± 2.95	The current study
Liquid cow manure	ZVI	0 10	108.86 ± 7.40 125.36 ± 3.98	
Residue	ZVI	0 10	67.56 ± 1.50 50.79 ± 3.52	
Starch	ZVI	0 10	349.21 ± 7.06 347.10 ± 14.35	
Microcrystalline cellulose	ZVI	0 10	193.25 ± 11.39 185.09 ± 8.90	
Waste activated sludge	ZVI	0 2 4 6 8 10 15	67.12 ± 1.77 126.17 ± 7.35 122.67 ± 0.57 137.62 ± 4.60 145.27 ± 0.64 160.02 ± 0.64 152.62 ± 0.21	
Organic fraction of municipal solid waste	ZVI	0 12	420 595	Kong et al., 2018
Waste activated sludge	ZVI	0 1 4 20	192.6 211.1 233.8 276.4	Feng et al., 2014
Lignin	FeCl_2_·4H_2_O	0 10 100	36 ± 1^a^ 29 ± 1^a^ 46 ± 1^a^	Wyman et al., 2019
Cellulose		010100	243 ± 1^a^ 239 ± 1^a^ 206 ± 1^a^	
Xylose		010100	101 ± 1^a^ 81 ± 1^a^ 41 ± 1^a^	
Rice straw	FeCl_3_·6H_2_O	0 3.22 ×10^−3^	264 263	Mancini et al., 2019

a *mL/g COD*.

By contrast, the main carbon source in the AD of cow manure and residue was crop straw, which was difficult to be degraded because of its complex lignocellulosic structure. According to the experimental results of this study in Table 2, ZVI increased the accumulative methane yield of liquid cow manure, but reduced the accumulative methane yield of cow manure and residue. It was concluded that Fe(II) and Fe(III) solids formed by ZVI were adsorbed on the surface of lignocellulose and reduced the available surface area of lignocellulose and the accessibility of cellulose, thereby inhibiting of the hydrolysis of lignocellulosic biomass. Besides, ZVI had potential risks of inhibition effect on bacterial activity, which could be attributed to the accumulation of solid iron particulates on the cell surface or overproduction of free radical species, thereby causing cellular injury (Wu et al., 2015). The detections of microbial community structure and enzyme activities offer a way to further explore the mechanism of ZVI inhibition in future studies. In conclusion, ZVI addition is not suitable for every AD system, which requires serious consideration.

## Conclusions

This study firstly investigated the effect of ZVI on the AD of cow manure. The accumulative methane and VFAs yield decreased by 10.3 and 12% with adding 10 g/L ZVI, respectively. ZVI decreased the methane yield of residue by 20.4%, while increased that of liquid cow manure by 15.2%, and did not change that of starch and microcrystalline cellulose. Experimental results indicated that ZVI promoted the AD of easily degradable organics, such as protein, lipid, and soluble carbohydrate, but inhibited the hydrolysis–acidification process of lignocellulose by reducing the available surface area and the accessibility of cellulose. With the increasing ISR, the effect of ZVI on methane production of cow manure changed from negative to positive. Further investigation indicated that the pretreatment of lignocellulosic biomass may be required before adding ZVI to enhance hydrolysis process, and then to increase methane yield.

## Data Availability Statement

The raw data supporting the conclusions of this article will be made available by the authors, without undue reservation.

## Author Contributions

ZL, LZha, XZ, and SC conceptualized the study. YM, XW, and WB completed the experiment and data analysis. YM, ZL, LZhe, and XW wrote and revised the manuscript. All authors listed have made a substantial, direct and intellectual contribution to the work, and approved it for publication.

## Conflict of Interest

The authors declare that the research was conducted in the absence of any commercial or financial relationships that could be construed as a potential conflict of interest.

## Abbreviations

AD: anaerobic digestion
BMP: biochemical methane potential
ISR: inoculum-substrate ratio
SBES: sodium 2-bromoethanesulfonate
TS: total solid
VS: volatile solid
VFAs: volatile fatty acids
ZVI: zero valent iron.
